# An Unusual Case of Genital Herpes Simplex Virus (HSV) Infection With Severe Non-necrotizing Soft Tissue Infection

**DOI:** 10.7759/cureus.82583

**Published:** 2025-04-19

**Authors:** Bung-Kook Ko, Xinyi Wei, Wei Ming Ong, Reizal Mohd Rosli, Alex Wong

**Affiliations:** 1 Colorectal Surgery, Eastern Health, Melbourne, AUS; 2 Medical School, Monash University, Melbourne, AUS

**Keywords:** anal ulcer, genital herpes, hsv-1, hsv-2, inguinal lymphadenopathy, inguinal node, inguinal swelling, perianal rash

## Abstract

Genital herpes simplex virus (HSV) infection is a prevalent condition with symptoms of genital ulcers, vesicles, or pustules, often along with malaise, fever, and inguinal lymphadenopathy. Although inguinal lymphadenopathy is quite common in genital HSV infection, it is uncommon to present without anogenital symptoms. This report highlights its clinical significance with a report of an immunocompetent 65-year-old male involved in high-risk sexual activities overseas who initially presented with only persistent inguinal lymphadenopathy. A perianal vesicular rash was identified on examination with features of anal trauma, as well as severe cellulitis without necrotizing features. The diagnosis of HSV-1 infection and the superimposed bacterial infection were confirmed with a swab of the rash and its serous discharge. We highlight an atypical presentation of anal HSV presenting as asymptomatic inguinal lymphadenopathy, and the importance of considering anal HSV in patient engaging in high-risk sexual behaviour when assessing a patient with inguinal lymphadenopathy.

## Introduction

Of more than 100 known herpes viruses, herpes simplex viruses (HSV) consist of two out of the eight that routinely infect humans only. They have a unique quad-layered structure consisting of an icosapentahedral capsid containing capsomers, surrounded by a protein coat called the tegument, and finally encased in a glycoprotein-lipid bilayer envelope. The HSV virus shares only 50% genomic homology and exhibits similarities in its mucocutaneous manifestations [1]. As transmission requires intimate contact, infections often affect the anal, vaginal, and oral regions, HSV infections may present as painful ulcers or blisters with fevers and malaise in the affected regions. Vesicular eruptions signify replication of the virus within the epithelial cells, followed by ascension along the sensory nerves to the dorsal root ganglia, where they establish latency. During reactivation, the virus spreads along the nerves from the ganglion to establish new mucocutaneous lesions. Interferon, humoral, mucosal, and cellular immunity play a crucial role in host defenses, and therefore, the severity of HSV infections increases in immunocompromised patients [1]. While in approximately 80% of cases, painful inguinal lymphadenopathy is observed, it is uncommon to be the sole manifestation of anal HSV infection and thus can complicate the diagnostic process ​[1-3]​. We present a unique case of HSV-1 in a previously healthy male, where the initial symptom was inguinal swelling that later progressed toward the perianal region, highlighting an uncommon presentation of this common infection.

## Case presentation

**Figure 1:**
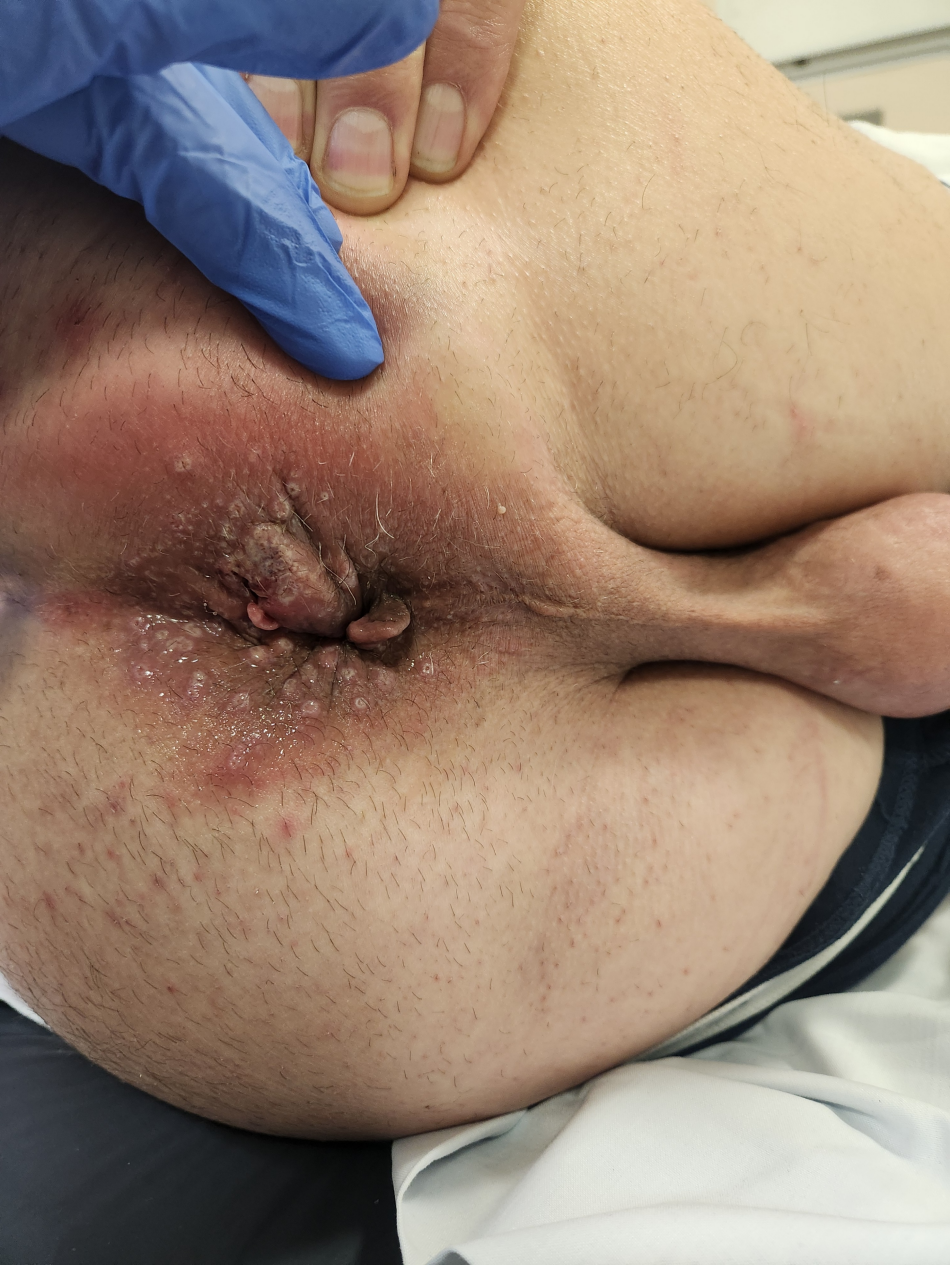
Perianal lump with adjacent ulcer and scattered vesicular rash with serous anal discharge.

**Figure 2:**
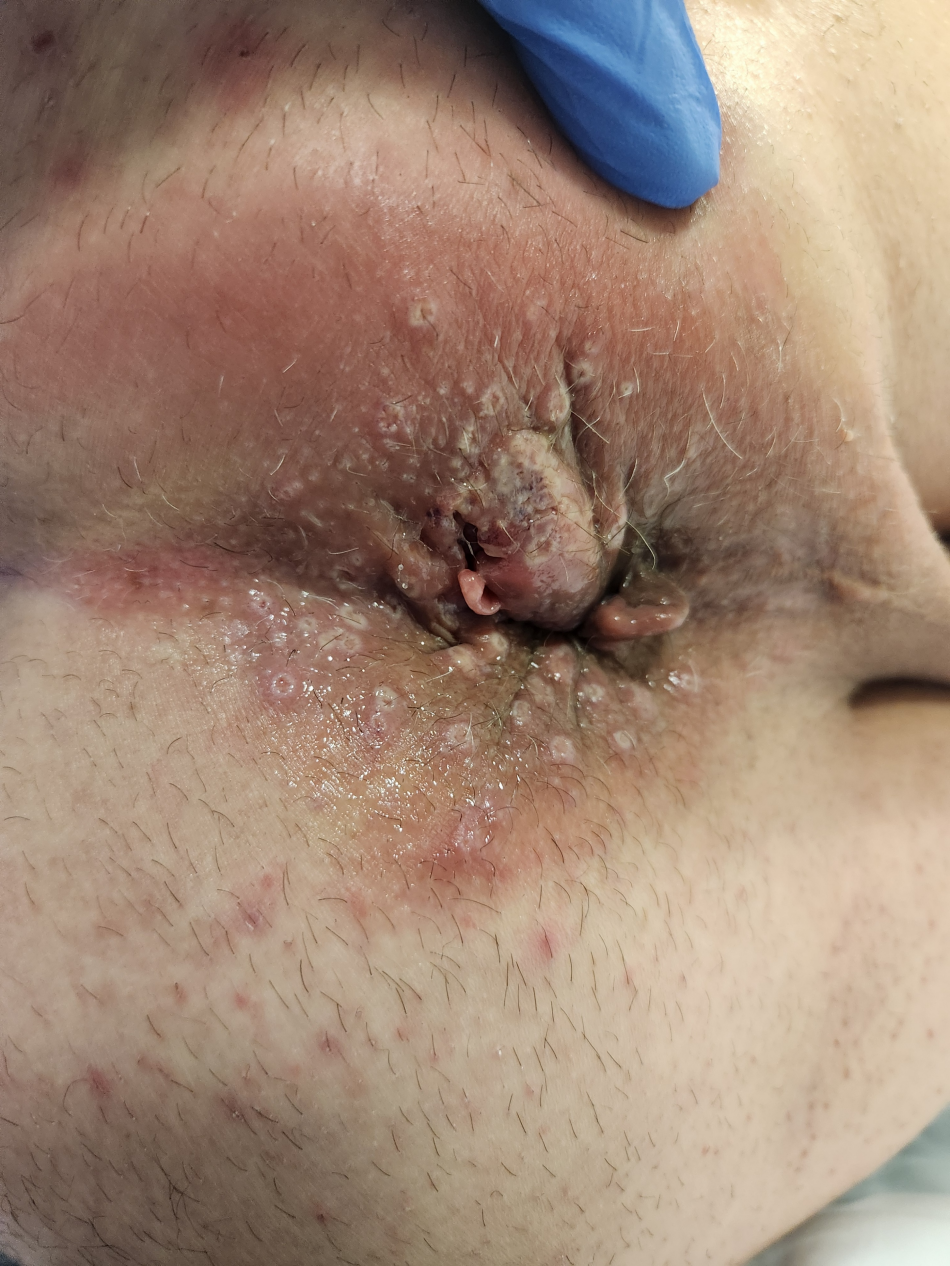
Perianal lump with adjacent ulcer and scattered vesicular rash with serous anal discharge.

**Figure 3:**
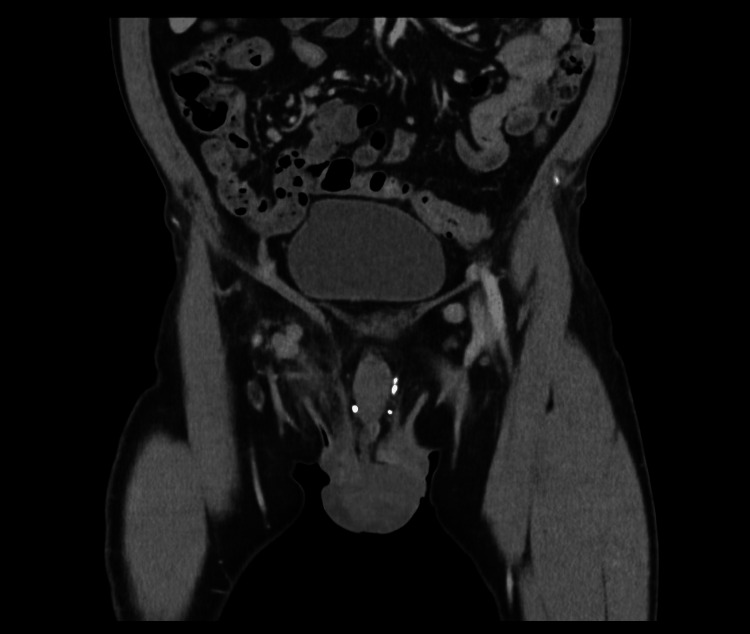
Computed tomography demonstrating soft tissue inflammation in the right groin with prominent inguinal lymph nodes.

**Figure 4:**
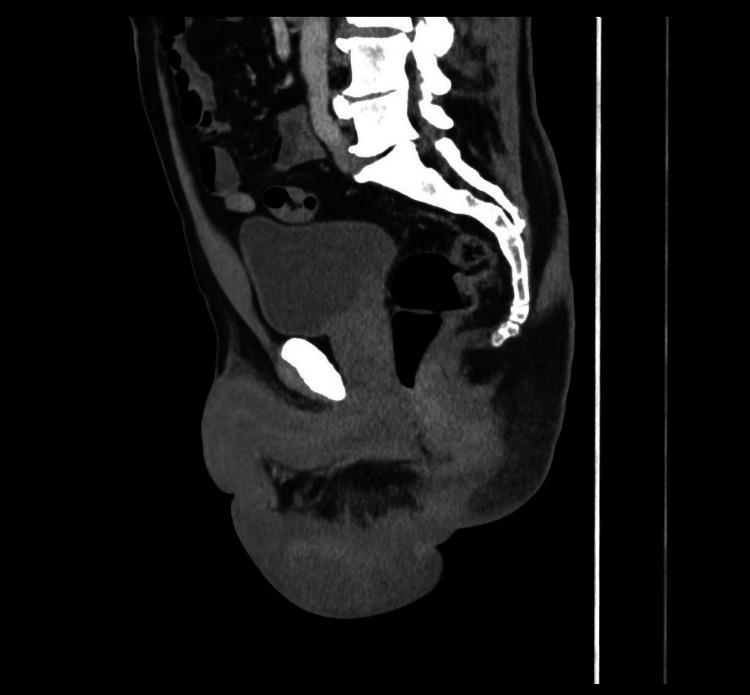
Computed tomography demonstrating soft tissue inflammation extending into the perineum.

A 65-year-old male presented with a four-week history of a right inguinal lump and concerns of necrotizing fasciitis. He initially noted the right inguinal lump four weeks before his ED presentation, with no other associated symptoms at the time. He was prescribed a trial of oral antibiotics with good resolution of symptoms. However, the right groin lump recurred a few weeks later, and the inguinal lump recurred, associated with rapid development of induration extending to the perineum and perianal region, accompanied by general malaise, fevers, and rigors. He has no significant past medical history. An ultrasound performed prior to his presentation stated a 24 mm hypoechoic area to the right of anus with vascularity showing possible abscess, notable thickening of the skin, and subcutaneous tissue to the right of the scrotum associated with tenderness and increased vascularity, raising concerns of necrotising fasciitis. On arrival, he was febrile with a temperature of 38.2°C but appeared well. Biochemical studies reflected a slightly elevated C-reactive protein (CRP) of 38.8 mg/L and white cell count of 7.9 x 109/L. Upon examination, he had a soft and non-tender abdomen. There was erythema and induration without crepitus along the right groin, extending to the right scrotum, perineum, perianal region, and right inferior gluteal fold. A right inguinal lump was palpable without an associated cough impulse. A firm and tender perianal lump was noted with adjacent skin tear at the anal verge and scattered vesicular rash surrounded the perianal region with serous discharge exuding from the anal canal (Fig. 1, Fig. 2). There were no internal masses identified on digital rectal examination. As the clinical picture was inconsistent with necrotizing fasciitis, he underwent a computed tomography scan, which demonstrated a stark inflammatory change within the soft tissues of the right groin, extending into the perineum with no gas formation with multiple prominent inguinal lymph nodes, concerning for severe cellulitis (Fig. 3, Fig. 4). A swab of the anal discharge returned positive for herpes HSV-1 and streptococcus pyogenes (Group A). No other sexually transmitted infections nor blood-borne viruses were identified. The patient subsequently disclosed that he had engaged in unprotected sexual intercourse overseas. Following consultation by the infectious diseases team, he was treated for anal herpes with superimposed bacterial cellulitis with intravenous benzylpenicillin and valaciclovir, and prompt resolution of cellulitis was observed. On follow-up, he achieved complete resolution of symptoms, and a colonoscopy at three months was normal. 

## Discussion

Genital HSV infection is a prevalent condition, impacting more than 13.3% of people aged 15-49 years worldwide, with HSV-1 accounting for approximately 70-80% and HSV-2 approximately 12-15% ​[2,4]​. HSV-1 first establishes primary infection in patients with no existing antibodies to either HSV subtypes. Non-primary initial infection is defined as infection with one HSV subtype in patients with existing antibodies to the other HSV type. HSV-1 transmission often results from contact with saliva or bodily fluids from an infected individual, and HSV-2 transmission often results from sexual contact with a seropositive individual during viral shedding ​[5,6]​. Historically, HSV-2 has been the predominant cause of genital herpes, whereas HSV-1 has primarily been associated with orolabial infections. However, due to an increased oral-genital intercourse, HSV-1 has become the leading cause of primary genital herpes ​[7,8]​. The virus proliferates at the infection site within the epithelium, causing vesicular eruptions, then travels to the dorsal root ganglia to establish latency until future reactivation ​[5]​. HSV possesses the ability to evade host defenses to successfully achieve latency. One of its many mechanisms involves inducing an intercellular accumulation and sequestering of CD1d molecules in antigen-presenting cells (APC). When CD1d molecules are transported to the cell surface of APC, it results in the stimulation of natural killer (NK) cells to eradicate the infected cells. However, when CD1d molecules are sequestered intercellularly, the APCs are unable to activate NK cells to eradicate HSV-1-infected cells. Subsequent periodic reactivation from latency, triggered by certain stimuli, can resume the lytic cycle [5]. The pathways for the HSV lytic cycle, latent phase, and reactivation follow distinct viral gene expression patterns, and the switch between pathways is controlled by complicated regulatory mechanisms involving numerous viral and host molecules [9]. 

Genital HSV usually causes recurrent, self-limited genital ulcers ​[10]​.  Greater than 50% of primary infections are associated with HSV-1 ​[4]​, while HSV-2 is the more frequent cause of recurrence ​[10]​. Up to 20% of seropositive HSV-1 persons will be asymptomatic ​[10]​, while up to 60% of new HSV-2 infections are ​[11]​. The presentation is usually associated with mild symptoms, does not always occur immediately after acquisition or exposure, and may be delayed by days or years ​[4]​. Incubation periods vary between one and 26 days, with a median of six to eight days ​[11]​. HSV-1 is usually associated with oral and genital lesions, while HSV-2 exclusively causes genital infections ​[10]​. Severe or frequent recurrences are managed with intermittent or suppressive antiviral therapy ​[4]​. Eighty percent of cases develop tender inguinal lymphadenopathy ​[11]​. 

Primary infection can present with vesicles, pustules, or extensive anogenital ulceration and nonspecific systemic features of fever, malaise, headache, and myalgias in up to 67% of cases ​[4,11]​. Lesions can also occur over the buttocks, thighs, pubis, and lower back ​[4]​ and are often erythematous, painful, and pruritic ​[4]​. Twenty percent of cases will present atypically ​[11]​. Other manifestations in the anogenital region include fissures, cervicitis, proctitis, urethritis, and very rarely, meningitis or transverse myelitis ​[4]​. 

While diagnosis is often made based on the clinical findings, confirmatory testing is performed by swabbing the base of the lesions for nucleic acid amplification testing, which has over 90% sensitivity and specificity ​[4,10]​. Histopathological features tissues with HSV infection demonstrate dense and deep infiltrates of lymphocytes near adnexal structures along with individual necrotic keratinocytes. Epidermal ballooning and acantholysis associated with vesicular eruptions are seen. Multinucleated giant cells and epithelial cells containing eosinophilic intranuclear inclusion bodies are seen on biopsies. There are no pathognomonic findings to differentiate HSV-1 from HSV-2 [5,6]. To our knowledge, only one case report of the diagnosis of HSV infection via excisional biopsy of the inguinal lymph node has been published. Histological examination of the lymph nodes exhibits florid follicular hyperplasia with attenuated mantle zones and adjacent monocytoid B-cell hyperplasia. Subsequent immunohistochemical stains of the lymph node biopsy are confirmatory for HSV in the two cases [3]. 

Current guideline management of HSV includes valaciclovir 500 mg BDS for five to 10 days or aciclovir 400 mg TDS for five to 10 days ​[4]​. When left untreated, symptoms typically last 16 days for a primary episode and nine to 11 days for recurrences ​[11]​. Opportunistic screening for STIs is also important, as co-infection in high-risk groups is common. Education should also be provided about safe sex practices such as condom use, pre-exposure prophylaxis (PrEP), and post-exposure prophylaxis (PEP) in high-risk populations and safe injecting practices ​[4]​. Vaccinations for HAV, HBV, and HPV should also be considered as indicated ​[4]​. Reactivation can be managed with episodic antivirals (short courses at the time of a recurrence) or long-term daily suppressive antivirals to prevent shedding and recurrence ​[4,10]​. Suppressive therapy can reduce the risk of transmission in men who have sex with men (MSM populations) ​[4,10]​. Suppressive therapy entails valaciclovir 500 mg ODS for six months versus self-initiated therapy at first signs of symptoms - valaciclovir 500 mg BDS for three days​ [4]​. Recurrences will occur within the first year after primary infection, in up to 90% of those with HSV-2 and 50% with HSV-1 ​[11]​. 

Atypical presentations of HSV can sometimes occur, particularly in immunocompromised individuals and, more rarely, in immunocompetent patients. Inguinal lymphadenopathy as the primary symptom of anal herpes is uncommon and can present as a diagnostic challenge, especially in immunocompetent individuals without prior history of genital lesions or known HSV infection ​[3]​. In rarer cases, generalized lymphadenopathy may also occur, mimicking infectious mononucleosis and lymphoma ​[12]​. 

This case demonstrates the importance of a comprehensive diagnostic approach, particularly when faced with atypical presentations. Microbiological testing, including PCR, viral cultures, or lesion swabs, is essential for confirming HSV infection. In genital HSV cases where typical genital lesions are absent, diagnosis could be made by lymph node biopsy and immunohistochemical staining ​[3]​. 

## Conclusions

HSV is a neurotropic virus with lytic and latent phases. It is a common infection with afflicted patients presenting with vesicular eruptions. Although infections are common in immunocompetent patients, immunosuppressed patients may experience severe morbidity from them. Although uncommon, HSV infection can also primarily present as inguinal lymphadenopathy without obvious genital or anal symptoms. Anal HSV or other perianal pathologies should be considered as a differential for inguinal lymphadenopathy, especially in patients with history of engaging in high-risk sexual behaviors. A thorough history to examine high-risk sexual behaviors and a detailed examination of the perineum and perianal region are crucial during the evaluation of a patient presenting with tender inguinal lymphadenopathy. We highlight the case of an unusual presentation of HSV infection with superimposed severe bacterial cellulitis, with the only presenting complaint as inguinal lymphadenopathy, and the patient was only forthcoming about his sexual history on prompting. Patients should be counselled on using barrier methods while engaging in sexual behaviors to reduce the risk of transmission and infection. 
